# Diagnostic Challenges due to a Germline Missense *MSH2* Variant in a Patient With Immunotherapy‐Responsive Locally Advanced Rectal Adenocarcinoma

**DOI:** 10.1002/cnr2.70037

**Published:** 2024-12-18

**Authors:** Gertruda Evaristo, Carla Harmath, Jeremy P. Segal, Ardaman Shergill, Namrata Setia

**Affiliations:** ^1^ Department of Pathology The University of Chicago Chicago Illinois USA; ^2^ Department of Radiology The University of Chicago Chicago Illinois USA; ^3^ Section of Hematology/Oncology, Department of Medicine The University of Chicago Chicago Illinois USA

**Keywords:** colorectal cancer, crc, immunotherapy, mmr, msi, rectal cancer

## Abstract

**Background:**

Rapid and accurate identification of mismatch repair (MMR) deficiency and Lynch syndrome is critical in the prognostication and clinical management of patients with colorectal carcinoma.

**Case Description:**

We describe here a young woman who developed a locally aggressive rectal adenocarcinoma with intact MMR protein expression by immunohistochemistry and absence of histologic evidence of MMR deficiency‐associated increased tumoral immune response. Germline DNA‐targeted sequencing identified *MSH2* variant p.R711P, initially classified as a variant of undetermined significance. Somatic tumoral DNA analysis revealed the identical *MSH2* variant, high tumor mutational burden, and microsatellite instability, in addition to superimposed alterations in *β2‐microglobulin* gene, possibly explaining the altered intratumoral immunity. Consequently, the patient was started on immunotherapy, leading to successful disease control (33 month follow‐up).

**Conclusion:**

The findings emphasize the utility of an integrative approach in the assessment of MMR status for determining candidacy for immunotherapy, especially in the setting of missense variants in MMR genes.

## 1 Introduction

DNA mismatch repair (MMR) system plays a critical role in maintaining genome integrity by identifying and correcting single‐base mismatches and short insertion and deletion loops that may occur during replication and recombination [1]. Defects in the key effectors of the MMR system lead to variation in short tandem DNA sequences termed microsatellites, that is microsatellite instability (MSI), and are observed in up to 25% of various sporadic tumors [2]. Germline alterations in MMR genes cause an autosomal dominant Lynch syndrome characterized by an increased risk of a wide spectrum of cancers, including colorectal, gynecological, upper gastrointestinal, and genitourinary cancers.

In humans, the MMR system comprises several heterodimers acting in a cascade of sequential steps. The mismatch recognition and initiation of repair is carried out primarily by MutSα complex, composed of MutS homologues 2 (MSH2) and 6 (MSH6) [3, 4, 5]. This complex then recruits the MutLα subunit, formed by MutL homologues MLH1 and PMS2, which orchestrates the mismatch excision and resynthesis. Several additional and redundant players, such as MSH3 and PMS1, but MLH1, MSH2, and MSH6, have been identified, and PMS2 represent the most common targets of MMR defects.

Recognition of these molecular events led to the development of germline multigene testing strategies for the diagnosis of Lynch syndrome patients [6, 7, 8]. Since population‐wide germline testing is not feasible, several cost‐effective screening algorithms have been additionally established to select the at‐risk patients and create preventive opportunities for them and their relatives, such as intensive surveillance and risk‐reducing surgeries. Central to the screening initiatives is the universal screening of colorectal and endometrial adenocarcinomas for MMR deficiency, which has become widely integrated within North American and European practice in the past decade [8, 9, 10]. The testing is most commonly performed by immunohistochemistry, using antibodies to MLH1, MSH2, MSH6, and PMS2, as this constitutes a rapid, relatively inexpensive, and readily accessible technique [11, 12]. Alternative methods focusing on the resultant MSI can be used, including polymerase chain reaction‐based detection of insertion/deletion mutations in a select group of microsatellite loci [13] or next‐generation sequencing (NGS)‐based assessment of all available microsatellite loci paired with quantification software such as MSI sensor [14]. However, the latter approaches are considerably more labour‐intensive and costly. In addition to guiding the genetic testing directly, the results of MMR/MSI testing can be integrated with clinical parameters into risk assessment models such as MMRpredict and MMRpro for further patient selection and prognostication [15, 16, 17].

Outside of the context of Lynch syndrome, MMR testing has a well‐established prognostic and predictive role in sporadic colorectal adenocarcinomas. The presence of MMR deficiency has been associated with better prognosis in *BRAF*‐wild type colorectal cancers but appears to reduce the benefit of fluorouracil‐based chemotherapy [18, 19]. More recently, MMR deficiency became an important predictor of response to PD‐1/PD‐L1 blockade [20, 21], further highlighting the clinical importance of MMR immunohistochemistry.

While loss of immunohistochemical reactivity can provide general information regarding the deficient protein, the specific alterations in MMR genes are more difficult to pinpoint given the underlying extensive molecular heterogeneity, with over 3000 unique germline sequence variants of MMR genes recorded in the International Society for Gastrointestinal Hereditary Tumors (InSiGHT) database (www.insight‐group.org; last accessed on August 25, 2024) [22, 23]. This adds to several thousands of reported variants of unknown significance (VUS), which further increases the complexity of efficient and accurate diagnosis of MMR deficiency. However, the precise diagnosis has important clinical, prognostic, and therapeutic implications, including indication for checkpoint inhibitor treatment [20, 21].

We report here a case of a young patient who presented with locally aggressive rectal adenocarcinoma, which demonstrated intact MMR proteins by immunohistochemistry. Subsequent demonstration of MSI by NGS‐based in‐house custom pipeline for MSI determination in the context of a unique germline missense *MSH2* variant led to successful disease control with immunotherapy.

## 2 Case Description

### 2.1 Clinical History

**FIGURE 1:**
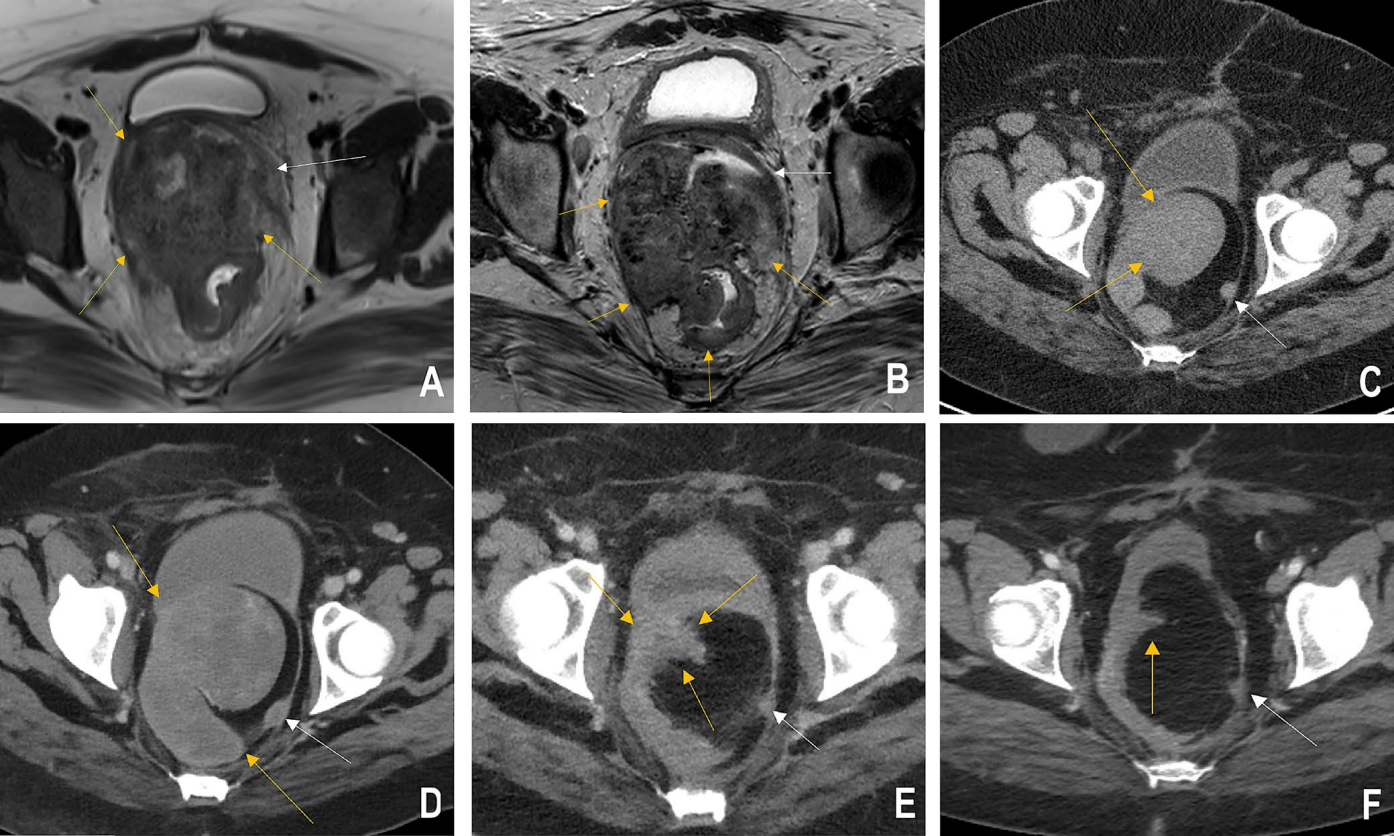
Preoperative and postoperative radiologic assessment of the rectal adenocarcinoma. (A, B) Preoperative Axial MRI T2WI demonstrates a large rectal mass with significant exophytic component, extending to the mesorectal fat and invading the mesorectal fascia (yellow arrows) and vagina (white arrows). (C, D) Postoperative axial CT exam of the pelvis with intravenous contrast reveals a recurrent mass (yellow arrows), with satellite nodule in the left pelvis (white arrow). (E, F) Post‐immunotherapy axial CT image of the pelvis with intravenous contrast shows a significant decrease in the mass following therapy. Small amount of residual soft tissue is present in the location of the recurrent mass seen on prior exam (yellow arrows) and a satellite nodule (white arrow).

A 35‐year‐old female patient presented to the University of Chicago Medical Center with a history of long‐standing pelvic pain and new onset rectal bleeding in February 2020. Colonoscopy demonstrated a large rectal mass which upon imaging revealed a 10‐cm tumor arising from upper/mid‐third of rectum, extending into vagina anteriorly and into pelvic floor muscle posteriorly (Figure 1A,B). The patient was treated with neoadjuvant capecitabine and concomitantly received 45 Gy radiation to the pelvis with a sequential rectal boost to 50.4 Gy at 1.8 Gy per fraction. She subsequently underwent a total proctocolectomy with en bloc removal of uterus, adnexa, and vagina, ileostomy reconstruction and neovagina reconstruction. The pathology revealed residual invasive rectal adenocarcinoma invading the vaginal wall, staged as ypT4bN0. There was no evidence of tumor regression (poor treatment response, score 3). The patient completed an adjuvant course of mFOLFOX with radiological evidence of remission, but within a month was discovered to have a neovaginal mass that was biopsy‐confirmed to be rectal adenocarcinoma (Figure 1C,D).

### 2.2 Pathologic Examination and Germline Sequencing Analysis

**FIGURE 2:**
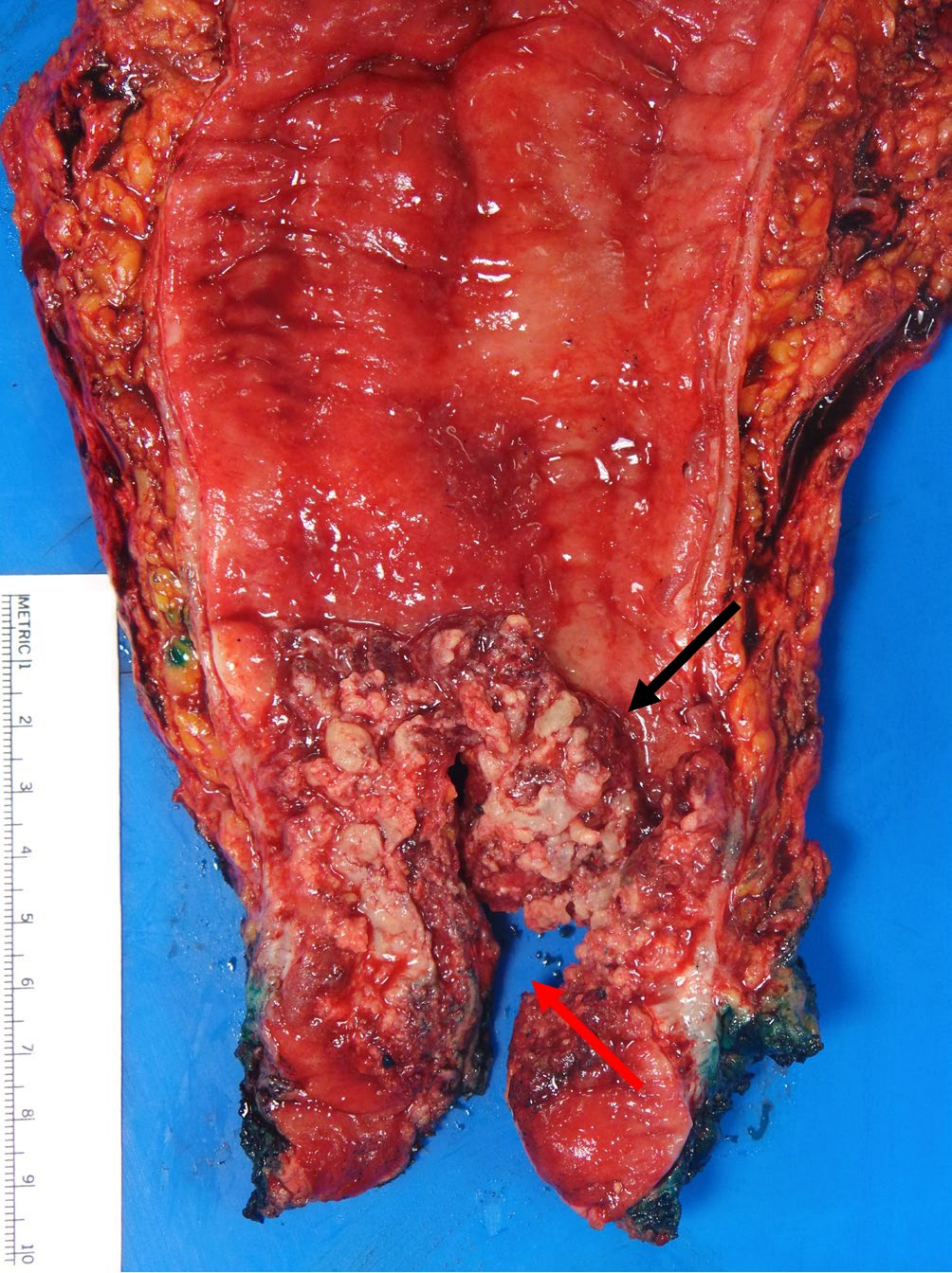
Macroscopic assessment of the rectal adenocarcinoma. Gross image of the resected portion of sigmoid colon with fragmented anus and vagina as part of total proctocolectomy with en‐bloc removal of uterus, adnexa, and vagina, showing a large friable tumor (black arrow) involving mid rectum with perforation through the tumor and invasion into the vaginal wall (red arrow).

**FIGURE 3:**
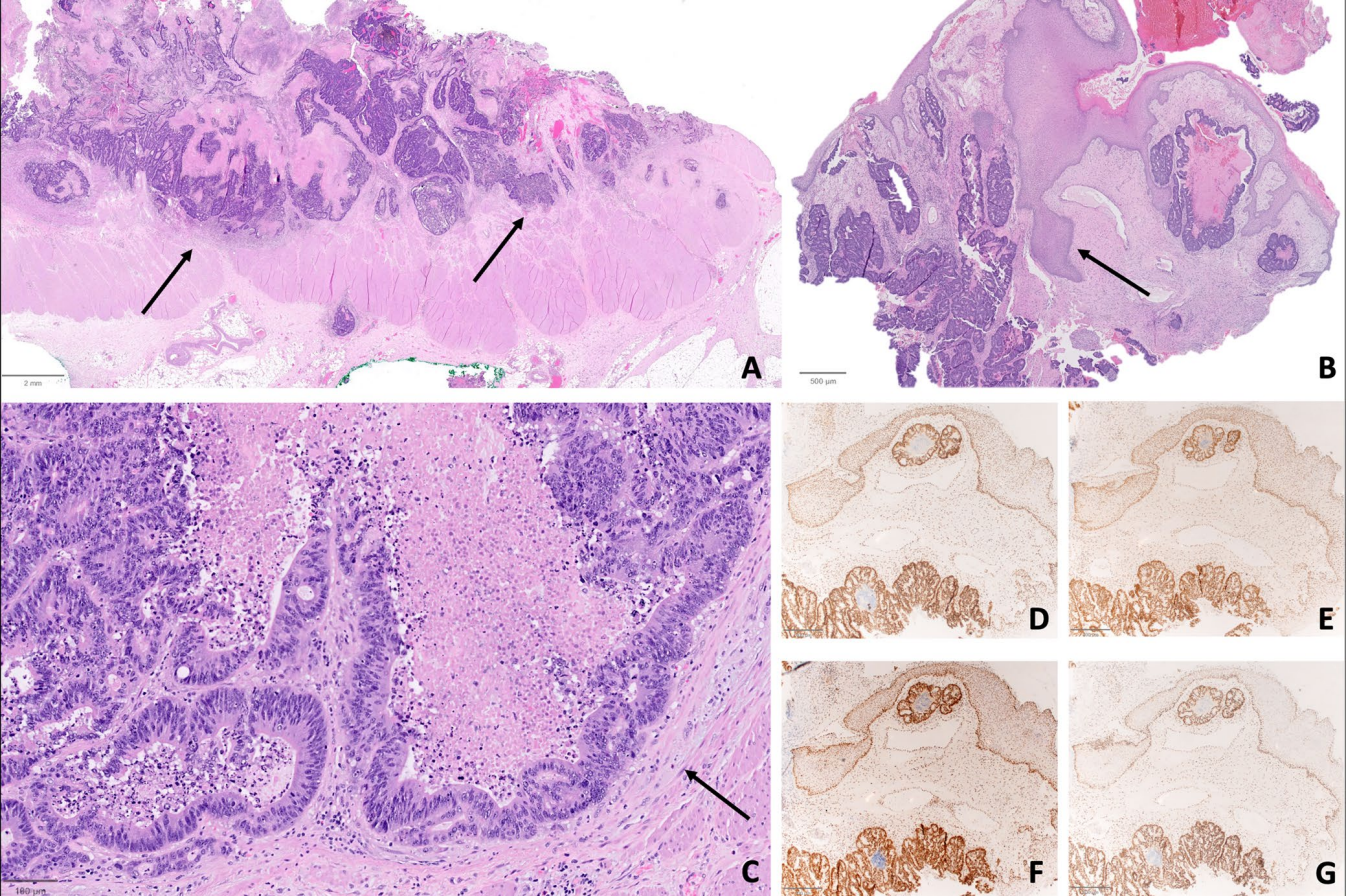
Microscopic assessment of the rectal adenocarcinoma. (A, B) The microscopic sections from the total proctocolectomy with en‐bloc removal of uterus, adnexa, and vagina show moderately differentiated colonic adenocarcinoma directly invading through the colonic wall (A, black arrow; original magnification 10×, scale bar = 2 mm) and into vagina (B, black arrow; original magnification 20×, scale bar = 500 um). The tumor shows no histologic features to suggest microsatellite instability, including mucin production, poorly differentiated adenocarcinoma, signet ring component, or Crohn's‐like lymphoid aggregates. (C) Higher magnification reveals the absence of tumor‐infiltrating lymphocytes in the peritumoral stroma (black arrow; original magnification 200×, scale bar = 100 um). (D–G) Immunohistochemistry for MLH1, MSH2, MSH6, and PMS2, respectively, shows intact nuclear staining (original magnification 20×, scale bar = 400 um).

The initial colonoscopic biopsies revealed a large rectal mass as well as a sigmoid colon polyp, which were diagnosed as a moderately differentiated rectal adenocarcinoma and a tubular adenoma, respectively. Immunostains for MMR proteins MLH1, MSH2, MSH6, and PMS2 all showed intact nuclear expression in adenocarcinoma cells. The resection specimen demonstrated a large rectal mass (Figure 2), which on microscopic examination revealed identical conventional adenocarcinoma morphology without any histologic features suggestive of MSI such as increased tumor‐infiltrating lymphocytes (TILs), Crohn‐like lymphoid reaction, poor differentiation or mucinous, signet ring, or medullary components (Figure 3A–C). The repeat MMR panel of MLH1, MSH2, MSH6, and PMS2 antibodies, reviewed by two gastrointestinal pathologists, was again intact (Figure 3D–G). The neovaginal mass was also confirmed to be rectal adenocarcinoma with conventional morphology without histologic features of MSI.

Given the unusual presentation and young age, the patient was referred to genetic counseling that revealed a maternal family history of multiple cancers, including colonic (two aunts, one uncle, grandfather, three great uncles), endometrial (mother, aunt, great aunt), brain (aunt, uncle), and pancreatic (great uncle) cancers and leukemia (aunt). Genomic DNA testing identified a heterozygous c.2132G > C missense mutation in exon 13 of *MSH2* gene, leading to a substitution of arginine with proline at codon 711. This alteration has been previously described in a Serbian family fulfilling revised Amsterdam criteria and was predicted to be deleterious in *in silico* analyses [24]. Nevertheless, the genomic DNA testing report classified the variant as a VUS at the time of the initial testing. The patient's report was subsequently amended, and the *MSH2* variant was reclassified as pathogenic. The amended report provided a generic comment for amendment without specific details.

### 2.3 Circulating Tumor DNA (ctDNA) and Somatic NGS Results

Given disease recurrence, the patient underwent a liquid biopsy to obtain further molecular characterization of her disease. The ctDNA analysis revealed an increased tumor mutational burden of 180.25 mutations/megabase, solidifying the possibility of a microsatellite unstable cancer. Concomitantly, the somatic mutational makeup of the neovaginal tumor was assessed with a targeted hybrid capture 155‐gene panel (Table 1). The *MSH2* c.2132G > C variant was redemonstrated, with allele frequency of 84%, consistent with a germline alteration and a loss of heterozygosity event. Most importantly, the tumor was confirmed to be microsatellite unstable with an associated tumor mutational burden of 45 mutations per megabase of interrogated genomic sequence. Additionally, two somatic mutations within *β2‐microglobulin* gene, c.244_247del and c.43_44dup, were identified. An essential component of MHC class I molecules, β2‐microglobulin plays a critical role in tumoral recognition by cytotoxic T cells [25] and could provide additional explanation for the lack of Lynch‐like histologic features in the current case.

**TABLE 1:** Select pathogenic alterations identified in the neovagina tumor.

Gene	Alteration	Variant allele frequency
*MSH2*	c.2132G > C, p.R711P (NM_000251.3)	84%
*APC*	c.471G > A, p.W157* (NM_000038.6)	33%
*ARID1A*	c.5548del, p.D1850Tfs*33 (NM_006015.6)	34%
*β2‐microglobuline*	c.244_247del, p.F82Ifs*20 (NM_004048.4)	31%
*β2‐microglobuline*	c.43_44dup, p.S16Ffs*29 (NM_004048.4)	35%
*PIK3CA*	c.328_330del, p.E110del (NM_006218.4)	34%
Ancillary findings		
Tumor mutational burden	45.0 mutations per megabase	
Microsatellite instability	Detected	

### 2.4 Treatment of Recurrent Disease and Follow‐Up

Based on the interim diagnosis of Lynch syndrome and demonstration of MSI, the patient's pelvic recurrence was treated with pembrolizumab monotherapy, which resulted in a marked reduction in pelvic disease (Figure 1E) and eventually a near complete radiographic response. Due to the toxicity of grade 3 arthralgias despite treatment, the immunotherapy was held after 54 weeks of therapy. The patient continued to have a relatively stable radiographic response as well as negative tumor‐informed molecular residual disease assay (Signatera) and tumor markers to date, a total of 33 months since initiation of immunotherapy and follow‐up. She has had no evidence of metastatic disease and no detectable ctDNA at 8, 11, 14, 17, 21, 24, and 27 months follow‐up.

## 3 Discussion

The present report provides a germline and somatic genetic characterization of a locally aggressive rectal adenocarcinoma in a young patient with germline *MSH2* p.R711P variant. Despite intact immunoexpression of MSH2 and other MMR proteins and lack of classical histologic evidence of increased immunologic response, NGS‐based identification of MSI in the setting of *MSH2* mutation allowed for accurate diagnosis and the appropriate patient selection for immunotherapy with pembrolizumab leading to sustained response.

While MMR deficiency has been widely established as the causative mechanism of Lynch syndrome, the precise underlying alterations in MMR genes display extensive molecular heterogeneity [22, 23]. Given the critical clinical implications, continuous effort is needed to further characterize the reported variants, particularly in instances where the diagnosis of Lynch is difficult to confirm, such as small families or atypical clinical presentations. As demonstrated by the current case, the clinical contextualization and the correct classification of *MSH2* p.R711P variant were critical for the appropriate diagnosis and treatment of this patient.

Like *MLH1* and *MSH6*, most *MSH2* alterations are truncating mutations, mainly nonsense or frameshift mutations [23]. The consequent loss of protein expression is at the basis for the utility of the immunohistochemistry as a quick and cost‐effective screening tool for MSI and Lynch syndrome. In the present case, however, despite several attempts on various tumoral tissues, the MMR immunohistochemistry failed to demonstrate MSH2 deficiency. False negative immunohistochemistry results usually occur in two settings. Firstly, MMR deficiency phenotype may uncommonly result from alterations in the additional players of the MMR machinery, such as MSH3 or PMS1, and therefore occur with intact MLH1, MSH2, MSH6, and PMS2 proteins. Another scenario, more applicable to the current case, is that of MMR deficiency resulting from an MMR gene alteration that leads to loss of function but preserved structure or antigenicity of the protein, as has been described in approximately 6% of microsatellite unstable cancers [26]. It is likely that the arginine to proline substitution at codon 711 does not interfere significantly with MSH2 protein production but rather affects its ABC‐ATPase domain spanning residues 620–855 of exons 7 through 13 [27]. Although the detailed mechanism remains controversial, the ATPase activity of MSH2 and its heterodimerization partner MSH6 is critical in the ability of MutSα to recognize mispaired or unpaired bases and recruit MutLα complex [5], supporting the pathogenic impact of this genomic alteration.

A compounding diagnostic challenge in the current case was the complete absence of classical morphologic features of Lynch syndrome, including TILs and Crohn‐like lymphoid aggregates. The identification of two pathogenic variants of *β2‐microglobulin* gene, c.244_247del and c.43_44dup, provides a potential explanation for this phenotype. The constant β2‐microglobulin light chain forms a complex with the variable heavy chain and antigen peptide to produce a functional MHC class I molecule that interacts with T‐cell receptor and activates cytotoxic T cells [12]. Interestingly, β2‐microglobulin gene contains several microsatellite repeats in its coding regions and is therefore prone to alterations in MMR‐deficient tumors, particularly small insertions and deletions as seen in the current case [28]. It is conceivable that β2‐microglobulin mutations would lead to the loss of a functional MHC class I molecule, which can in turn modulate tumoral antigenicity, immune microenvironment and/or T cell response, leading to loss of the classical Lynch‐associated histologic features. This concept has been proposed to explain worse prognosis and poor response to immunotherapy seen in association with *β2‐microglobulin* gene alterations in some studies [25, 29]. However, other authors have demonstrated positive immunotherapy outcomes in *β2‐microglobulin*‐mutated cases, as seen in the current patient, through proposed mechanisms such as activation of CD4^+^ T cell‐predominant response and retention of MHC class I molecules [30, 31]. This emphasizes the complexity of the interacting factors in cancer immunogenicity and immunotherapy and highlights the need for further characterization of factors predictive of checkpoint inhibitor response.

Its localization within the highly conserved ABC‐ATPase domain, its association with the clinical features of Lynch syndrome in the current patient's case and a previously described family [24], and the segregation studies are all in support of the pathogenic nature of *MSH2* p.R711P variant. However, the main limitation in the present report remains the absence of functional studies confirming the deleterious effect of this alteration. This is currently palliated by bioinformatics algorithms unanimously predicting a pathogenic impact on protein structure and function [24], but future work will be needed to confirm the biological effect of this alteration.

In conclusion, we presented a case of a young woman with locally advanced rectal adenocarcinoma with no histologic or immunohistochemical evidence of MMR deficiency but with NGS‐based identification of MSI in the setting of a missense *MSH2* variant that allowed for appropriate immunotherapy with a sustained response. Beyond this specific variant, this report highlights the importance of an integrative, multimodality approach to the diagnosis of MMR deficiency, which is essential for the appropriate recognition of syndromic patients and accurate prognostic and therapeutic guidance.

## Author Contributions

**Gertruda Evaristo:** conceptualization, writing – original draft, methodology, data curation, formal analysis, writing – review and editing. **Carla Harmath:** data curation, writing – review and editing. **Jeremy P. Segal:** data curation, writing – review and editing. **Ardaman Shergill:** data curation, writing – review and editing. **Namrata Setia:** conceptualization, methodology, formal analysis, data curation, writing – original draft, writing – review and editing, supervision.

## Ethics Statement

The study was reviewed by the University of Chicago institutional Review Boards and obtained exemption from ethics approval based on case report nature.

## Consent

The patient's informed consent to genomic investigations, analysis, and publication of results was obtained.

## Conflicts of Interest

The authors declare no conflicts of interest.

## Data Availability

The data that support the findings of this study are available from the corresponding author upon reasonable request.

## References

[1] R. R. Iyer , A. Pluciennik , V. Burdett , and P. L. Modrich , “DNA Mismatch Repair: Functions and Mechanisms,” Chemical Reviews 106 (2006): 302–323. 16464007 10.1021/cr0404794

[2] P. Peltomäki , “Role of DNA Mismatch Repair Defects in the Pathogenesis of Human Cancer,” Journal of Clinical Oncology 21 (2003): 1174–1179. 12637487 10.1200/JCO.2003.04.060

[3] T. M. Marti , C. Kunz , and O. Fleck , “DNA Mismatch Repair and Mutation Avoidance Pathways,” Journal of Cellular Physiology 191 (2002): 28–41. 11920679 10.1002/jcp.10077

[4] A. Bellacosa , “Functional Interactions and Signaling Properties of Mammalian DNA Mismatch Repair Proteins,” Cell Death and Differentiation 8 (2001): 1076–1092. 11687886 10.1038/sj.cdd.4400948

[5] M. H. Lamers , H. H. Winterwerp , and T. K. Sixma , “The Alternating ATPase Domains of MutS Control DNA Mismatch Repair,” EMBO Journal 22 (2003): 746–756. 12554674 10.1093/emboj/cdg064 PMC140748

[6] D. Georgiou , L. Monje‐Garcia , T. Miles , K. Monahan , and N. A. J. Ryan , “A Focused Clinical Review of Lynch Syndrome,” Cancer Management and Research 15 (2023): 67–85. 36699114 10.2147/CMAR.S283668 PMC9868283

[7] N. M. Lindor , G. M. Petersen , D. W. Hadley , et al., “Recommendations for the Care of Individuals With an Inherited Predisposition to Lynch Syndrome: A Systematic Review,” Journal of the American Medical Association 296 (2006): 1507–1517. 17003399 10.1001/jama.296.12.1507

[8] Evaluation of Genomic Applications in Practice and Prevention (EGAPP) Working Group , “Recommendations From the EGAPP Working Group: Genetic Testing Strategies in Newly Diagnosed Individuals With Colorectal Cancer Aimed at Reducing Morbidity and Mortality From Lynch Syndrome in Relatives,” Genetics in Medicine 11 (2009): 35–41. 19125126 10.1097/GIM.0b013e31818fa2ff PMC2743612

[9] F. M. Giardiello , J. I. Allen , J. E. Axilbund , et al., “Guidelines on Genetic Evaluation and Management of Lynch Syndrome: A Consensus Statement by the US Multi‐Society Task Force on Colorectal Cancer,” Gastroenterology 147 (2014): 502–526. 25043945 10.1053/j.gastro.2014.04.001

[10] S. Gupta , D. Provenzale , X. Llor , et al., “Genetic/Familial High‐Risk Assessment: Colorectal,” Journal of the National Comprehensive Cancer Network 17, no. 9 (2019): 1032–1041. 31487681 10.6004/jnccn.2019.0044

[11] H. Hampel , W. L. Frankel , E. Martin , et al., “Feasibility of Screening for Lynch Syndrome Among Patients With Colorectal Cancer,” Journal of Clinical Oncology 26 (2008): 5783–5788. 18809606 10.1200/JCO.2008.17.5950 PMC2645108

[12] J. F. Hechtman , S. Middha , Z. K. Stadler , et al., “Universal Screening for Microsatellite Instability in Colorectal Cancer in the Clinical Genomics Era: New Recommendations, Methods, and Considerations,” Familial Cancer 16 (2017): 525–529. 28405781 10.1007/s10689-017-9993-x

[13] C. R. Boland , S. N. Thibodeau , S. R. Hamilton , et al., “A National Cancer Institute Workshop on Microsatellite Instability for Cancer Detection and Familial Predisposition: Development of International Criteria for the Determination of Microsatellite Instability in Colorectal Cancer,” Cancer Research 58 (1998): 5248–5257. 9823339

[14] B. Niu , K. Ye , Q. Zhang , et al., “MSIsensor: Microsatellite Instability Detection Using Paired Tumor‐Normal Sequence Data,” Bioinformatics 30 (2014): 1015–1016. 24371154 10.1093/bioinformatics/btt755 PMC3967115

[15] R. A. Barnetson , A. Tenesa , S. M. Farrington , et al., “Identification and Survival of Carriers of Mutations in DNA Mismatch‐Repair Genes in Colon Cancer,” New England Journal of Medicine 354 (2006): 2751–2763. 16807412 10.1056/NEJMoa053493

[16] S. Chen , W. Wang , S. Lee , et al., “Prediction of Germline Mutations and Cancer Risk in the Lynch Syndrome,” Journal of the American Medical Association 296 (2006): 1479–1487. 17003396 10.1001/jama.296.12.1479 PMC2538673

[17] R. C. Green , P. S. Parfrey , M. O. Woods , and H. B. Younghusband , “Prediction of Lynch Syndrome in Consecutive Patients With Colorectal Cancer,” Journal of the National Cancer Institute 101 (2009): 331–340. 19244167 10.1093/jnci/djn499

[18] C. M. Ribic , D. J. Sargent , M. J. Moore , et al., “Tumor Microsatellite‐Instability Status as a Predictor of Benefit From Fluorouracil‐Based Adjuvant Chemotherapy for Colon Cancer,” New England Journal of Medicine 349 (2003): 247–257. 12867608 10.1056/NEJMoa022289 PMC3584639

[19] P. Lochhead , A. Kuchiba , Y. Imamura , et al., “Microsatellite Instability and BRAF Mutation Testing in Colorectal Cancer Prognostication,” Journal of the National Cancer Institute 105 (2013): 1151–1156. 23878352 10.1093/jnci/djt173 PMC3735463

[20] D. Le , J. Uram , H. Wang , et al., “PD‐1 Blockade in Tumors With Mismatch‐Repair Deficiency,” New England Journal of Medicine 372 (2015): 2509–2520. 26028255 10.1056/NEJMoa1500596 PMC4481136

[21] D. Le , J. Durham , K. Smith , et al., “Mismatch Repair Deficiency Predicts Response of Solid Tumors to PD‐1 Blockade,” Science 357 (2017): 409–413. 28596308 10.1126/science.aan6733 PMC5576142

[22] B. A. Thompson , A. B. Spurdle , J. P. Plazzer , et al., “Application of a 5‐Tiered Scheme for Standardized Classification of 2,360 Unique Mismatch Repair Gene Variants in the InSiGHT Locus‐Specific Database,” Nature Genetics 46 (2014): 107–115. 24362816 10.1038/ng.2854 PMC4294709

[23] P. Peltomäki , “Update on Lynch Syndrome Genomics,” Familial Cancer 15 (2016): 385–393. 26873718 10.1007/s10689-016-9882-8 PMC4901089

[24] G. Thodi , F. Fostira , R. Sandaltzopoulos , et al., “Screening of the DNA Mismatch Repair Genes MLH1, MSH2 and MSH6 in a Greek Cohort of Lynch Syndrome Suspected Families,” BMC Cancer 10 (2010): 544. 20937110 10.1186/1471-2407-10-544 PMC2976752

[25] M. Bernal , F. Ruiz‐Cabello , A. Concha , A. Paschen , and F. Garrido , “Implication of the β2‐Microglobulin Gene in the Generation of Tumor Escape Phenotypes,” Cancer Immunology, Immunotherapy 61 (2012): 1359–1371. 22833104 10.1007/s00262-012-1321-6 PMC11029609

[26] J. F. Hechtman , S. Rana , S. Middha , et al., “Retained Mismatch Repair Protein Expression Occurs in Approximately 6% of Microsatellite Instability‐High Cancers and Is Associated With Missense Mutations in Mismatch Repair Genes,” Modern Pathology 33 (2020): 871–879. 31857677 10.1038/s41379-019-0414-6 PMC7195218

[27] J. J. Warren , T. J. Pohlhaus , A. Changela , R. R. Iyer , P. L. Modrich , and L. S. Beese , “Structure of the Human MutSalpha DNA Lesion Recognition Complex,” Molecular Cell 26 (2007): 579–592. 17531815 10.1016/j.molcel.2007.04.018

[28] F. Liu , F. Zhong , H. Wu , et al., “Prevalence and Associations of Beta2‐Microglobulin Mutations in MSI‐H/dMMR Cancers,” Oncologist 28 (2023): e136–e144. 36724040 10.1093/oncolo/oyac268 PMC10020813

[29] S. Yeon Yeon , S. H. Jung , Y. S. Jo , et al., “Immune Checkpoint Blockade Resistance‐Related B2M Hotspot Mutations in Microsatellite‐Unstable Colorectal Carcinoma,” Pathology, Research and Practice 215 (2019): 209–214. 30503610 10.1016/j.prp.2018.11.014

[30] G. Germano , S. Lu , G. Rospo , et al., “CD4 T Cell‐Dependent Rejection of Beta‐2 Microglobulin Null Mismatch Repair‐Deficient Tumors,” Cancer Discovery 11 (2021): 1844–1859. 33653693 10.1158/2159-8290.CD-20-0987

[31] S. Middha , R. Yaeger , J. Shia , et al., “Majority of B2M‐Mutant and ‐Deficient Colorectal Carcinomas Achieve Clinical Benefit From Immune Checkpoint Inhibitor Therapy and Are Microsatellite Instability‐High,” JCO Precis Oncologia 3 (2019): 1–14. 10.1200/PO.18.00321 PMC6469719 31008436

